# 

**Published:** 1894-07-14

**Authors:** 


CONTENTS.

The Summits of Medical Progress - - - - - - - 309
Around the Hospitals - - - - - - - - - - - - - 310
The Role of Bromine in Therapeutics and Bromal Hydrate. By Sir Benjamin Ward Richardson, M.D., F.R.S. 311
Some Aspects of Medical Science - - - - - - - 312
The First Century of English Medical Literature.—VII. The Secrets of Alexis - - - - - - - - - - 313
Medical Progress and Hospital Clinics.—The Treatment of Angular Deformity of the Spine, or Spinal Caries - - - 317
Diseases of the Ear.—III. - - - - - - - - - - 319
Medical Progress.—Scarlet Fever - - - - - - - 320
New Drugs and Preparations - - - - - - - - - 321
New Appliances and Things Medical - - - - - - 321
Editors' Letter-Box - - - - - - - - - - - - - 322
Studies in Applied Sociology.—XII. How Workers Live—The Cotton Industry - - - - - - - - - - - - - - 314
Annotations.—Is Cure Superfluous?—The Bacillus Diphtheriæ in Cows' Milk—Derby Sets an Example - - - - - - 315
Notes and News - - - - - - - - - - - - - - - 316
The Institutional Workshop:
Story of the Insane from Year to Year - - - - - 323
Hospital Construction.—The New Derbyshire Royal Infirmary 325
Practical Departments - - - - - - - - - - - 325
Practical Points - - - - - - - - - - - - - - 325
Hospital Festivals and Meetings - - - - - - - 325
Construction Notes - - - - - - - - - - - - - 326
Scraps and Gleanings - - - - - - - - - - - - 327
The Book World of Medicine and Science - - - - 327

EXTRA SUPPLEMENT.—THE NURSING MIRROR.

News from the Nursing World.—The Royal Christening—Princess Beatrice and the Queen's Nurses—Violence Towards a Pauper—The Attractions of Lawn Tennis—Sick Paupers at Bath—Timely Advice—Compulsory Prayers—Hidden Perils—Queen's Nurse at Drogheda—"Sisters of the People"—Our Soldiers' Wives—Ophthalmia and Eczema—Nurses for Ceylon—Steinhousemuir—Royal Infirmary, Derby—Short Items - - cli
On General Nursing.—XX. Peritonitis (*continued*) - - cliii
The Mechanism of Movement.—VIII. The Hand - - cliv
Our American Letter—Asylum Notes—Ireland - - clv
Notes from Germany—Our Examinations - - - - clv
Imagination and Emotion - - - - - - - - clvi
White Phosphorus and Necrosis - - - - - - clvii
The National Home Reading Association - - - clvii
Women's Work.—Health Lecturing in Rural Districts - clviii
Queen Victoria's Jubilee Institute for Nurses - - clviii
A Case for Assistance—Notes and Queries - - - clviii
Our American Sisters - - - - - - - - - - clix
Appointments—Minor Appointments - - - - - clix
Everybody's Opinion—For Reading to the Sick - - clx

